# Optimizing the roles of health workers to improve access to health services in Africa: an implementation framework for task shifting and sharing for policy and practice

**DOI:** 10.1186/s12913-023-09848-z

**Published:** 2023-08-09

**Authors:** Sunny C Okoroafor, Christmal Dela Christmals

**Affiliations:** 1 Universal Health Coverage - Life Course Cluster, World Health Organization Country Office for Uganda, Kampala, Uganda; 2 Centre for Health Professions Education, Faculty of Health Sciences, North-West University, Potchefstroom Campus, Potchefstroom, South Africa

**Keywords:** Implementation framework, Task shifting, Task sharing, Africa, Health services, Health workers

## Abstract

**Background:**

Globally, countries are taking actions to ensure that their population have improved access to people-centred and integrated health services. Attaining this requires improved access to health workers at all levels of health service delivery and equitably distributed by geographical location. Due to the persistent health worker shortages, countries have resorted to implementing task shifting and task sharing in various settings to optimally utilize existing health workers to improve access to health services. There are deliberations on the need for an implementation framework to guide the adoption and operationalization of task shifting and task sharing as a key strategy for optimally utilizing the existing health workforce towards the achievement of UHC. The objective of this study was to develop an implementation framework for task shifting and task sharing for policy and practice in Africa.

**Methods:**

A sequential multimethod research design supported by scoping reviews, and qualitative descriptive study was employed in this study. The evidence generated was synthesized into an implementation framework that was evaluated for applicability in Africa by 36 subject matter experts.

**Results:**

The implementation framework for task shifting and task sharing has three core components – context, implementation strategies and intended change. The implementation strategies comprise of iterative actions in the development, translation, and sustainment phases that to achieve an intended change. The implementation strategies in the framework include mapping and engagement of stakeholders, generating evidence, development, implementation and review of a road map (or action plan) and national and/or sub-national policies and strategies, education of health workers using manuals, job aids, curriculum and clinical guidelines, and monitoring, evaluation, reviews and learning.

**Conclusion:**

The implementation framework for task shifting and task sharing in Africa serves as a guide on actions needed to achieve national, regional and global goals based on contextual evidence. The framework illustrates the rationale and the role of a combination of factors (enablers and barriers) in influencing the implementation of task shifting and task sharing in Africa.

**Supplementary Information:**

The online version contains supplementary material available at 10.1186/s12913-023-09848-z.

## Introduction

Globally, countries are taking actions to ensure that their population have improved access to people-centred and integrated health services [1]. Accomplishing this is vital in realizing universal health coverage (UHC) which is a target of the sustainable development goals (SDG) [2]. Attaining this requires improved access to a suitable skill-mix of health workers that are qualified, skilled and competent at all levels of health service delivery and equitably distributed by geographical location [3]. However, slow progress is being made in this regard due to prevalent global health worker shortages which is projected to be 15 million by 2020 and 10 million by 2030 based on current trends [4]. For Africa, the projected shortfall is more than 5 million and it contributes significantly to the projected global shortfall [4].

To ameliorate the impact of these shortages, countries have resorted to implementing task shifting and task sharing in various settings to optimally utilize existing health workers to improve access to health services, including expanding the delivery of services within certain contexts [5, 6]. Task shifting and task sharing is defined as the reallocation of tasks within health worker groups from trained and qualified health workers to other health workers with shorter training duration towards maximizing the available health workforce [5]. In task shifting, tasks are delegated or transferred, and in task sharing, tasks are delivered collaboratively by different staff categories [7]. In Africa, task shifting and task sharing have been implemented in various health services context including hypertension, tuberculosis, reproductive, maternal and newborn health, child and adolescent health, HIV/AIDS, tuberculosis, and mental health [8–12]. To enhance the capacities of health workers to deliver shifted or shared tasks, various health professions education strategies have been applied, including preservice and in-service education, clinical mentoring, episodic supportive supervision, and provision of job aids [13–15].

With the extensive application of task shifting and task sharing, there are deliberations on the need for an implementation framework to guide the adoption and operationalization of task shifting and task sharing as a key strategy for optimally utilizing the existing health workforce towards the achievement of UHC. This is because there is a persistent fragmented approach to its implementation, and a weak integration of implementation strategies being applied into national policies, guidelines and programmes. In addition, there remains a gap between adapting global, regional and national evidence on task shifting and task sharing research into practice, and this is contributing to the slow achievement of the set objectives. The development of frameworks is recommended to fill this gap as they guide the translation of evidence into practice to facilitate maximal utilization of health research in policy and practice by policymakers rather than the current trend of being guided by uninformed political considerations [16, 17]. Specifically, implementation frameworks are being promoted considering that it considers steps or process or stages of implementation, areas or levels of influence and the elements of factors/ determinants i.e., barriers and promoters of practice, strategies/approaches to mitigate the impact of the factors and implement the best practice, and evaluations/ assessment of impact [18, 19]. The objective of this study was to develop an implementation framework for task shifting and task sharing for policy and practice in Africa.

## Methods

### Study design

A sequential multimethod research design supported by scoping reviews and qualitative descriptive study was employed in this study. There were four key phases before the dissemination of the study findings. These phases were informed by steps for framework design and development [20]. In phase 1, a scoping review was conducted to synthesize evidence on the rationale and scope of task shifting and task sharing in Africa, and the health professions education strategies applied to enhance capacities for task shifting and task sharing implementation in Africa. In phase 2, a qualitative study exploring the perceptions of policymakers on the barriers, promoters, and strategies for improving task shifting and task sharing implementation in Nigeria was conducted. Subsequently, evidence generated from the scoping review, and the qualitative explorative-descriptive study was synthesized and used to design and develop an implementation framework (phase 3). In phase 4, subject matter experts evaluated the applicability of the framework in Africa.

### Data collection and analysis

#### Scoping reviews

Two scoping reviews were conducted using the enhanced Arksey and O’Malley’s framework for scoping reviews [21, 22]. The scoping review was conducted in five steps: (1) identify research questions, (2) identify appropriate studies, (3) select relevant studies, (4) extract and chart data and (5) summarise and report results. The scoping reviews focused on answering the following questions: (1) What are the documented rationales for task shifting and task sharing, and the scope of tasks shifted or shared to improve access of the population to health services in Africa? and (2) What health professions education strategies have been applied to enhance health workers’ capacities for task shifting and task sharing in Africa? PubMed, Scopus and CINAHL bibliographic databases were searched to obtain peer-reviewed papers, specifically quantitative, qualitative, and mixed-methods studies, as well as review and perspective papers on task shifting or sharing for integrated health service delivery. A data matrix was used to extract data from studies applying the thematic analysis approach [23, 24]. The reviews were reported using the Preferred Reporting Items for Systematic Reviews and Meta-Analyses guidelines Extension for Scoping Review reporting standards [23, 24].

#### Qualitative study

An explorative descriptive qualitative study exploring perceptions of policymakers on barriers and promoters of task shifting and sharing, and strategies for improving task shifting and task sharing implementation in Nigeria was conducted. Purposive and snowball sampling techniques [25] were applied in selecting a sample of 20 policymakers in Bauchi and Cross River States. The purposeful sampling targeted stakeholders involved in policy formulation and/or implementation at sub-national levels. Bauchi and Cross River States were purposively selected for this study because of their experience in adapting and implementing task shifting and task sharing policies. Key informant in-person interviews were conducted using a semi-structured interview guide with data collected using field note-taking and audio recording. The audio-recorded information was transcribed verbatim and analysed using the thematic analysis approach [26, 27] with the Nvivo 12 Pro software.

#### Develop and evaluate the implementation framework

The findings of the scoping reviews and the qualitative study were synthesized [28] into an implementation framework. The target population for the evaluation phase were subject matter experts on task shifting and sharing in Africa purposefully selected based on the publications obtained during the scoping review process and the policymakers interviewed in Nigeria during the qualitative phase. Thirty-six (36) purposefully selected subject matter experts evaluated the implementation framework using a template exploring their views on context, applicability and how the framework can be improved (Supplementary file 1). The template was peer-reviewed by experts in implementation research and scholars in teaching and learning. The experts were sent an online invitation with 57% of the experts who were willing to participate indicating their informed consent by clicking on a link to a QuestionPro form where the template had been uploaded. The completed templates were analysed by comparing suggestions and synthesizing the views into the final implementation framework.

#### Ethics approval

The North-West University Health Research Ethics Committee (NWU-00099-22-A1, 31 August 2022) approved this study. The qualitative component was approved by the National Health Research Ethics Committee (NHREC) of Nigeria’s Federal Ministry of Health (NHREC/01/01/2007-30/06/2022). Informed consent was obtained from all study respondents.

## Results

### Scoping review on rationale and scope of task shifting and task sharing implementation in Africa

Sixty-one papers were included in the final review. The rationale for task shifting and task sharing were shortages in health workers, ensuring optimal use of current health workers and expanding access to health services. The shortage of health workers was marked in rural areas and by levels of health care service delivery and these also informed the need to expand health services. the scope of tasks shifted and/or shared in 23 countries included maternal and child health, surgical care, medicines management, sexual and reproductive health, communicable diseases (tuberculosis and HIV/AIDS), NCD (hypertension, diabetes, mental health, and eyecare),, medicines management and emergency care [29].

### Scoping review on health professions education strategies for enhancing capacity for task shifting and task sharing implementation in Africa

Thirty eight studies from 23 countries were included in the study. The health professions education strategies implemented were education (preservice and inservice), clinical supervision and mentoring, supportive supervision, and providing job aides. The health services contexts where these strategies were applied include various health services contexts including general health, cancer screenings, reproductive, maternal, newborn, child and adolescent health, HIV/AIDS, emergency care, hypertension, tuberculosis, eye care, diabetes, mental health, and medicines. The findings indicate the importance of education based on curriculum and job aids in improving the knowledge and skills of health workers, and the need for their being informed by a needs assessment. Furthermore, clinical supervision and mentoring, and supportive supervision should be informed by protocols and service guidelines based on contextual characteristics [30].

### Qualitative study on barriers, promoters, and strategies for improving task shifting and task sharing implementation

Addressing the shortage of health workers to deliver health services was reported as the rationale for task shifting and task sharing implementation in Nigeria. Persistent shortage of health workers, inter-cadre rivalry, the perceived sub-optimal capacity of the beneficiary cadres of task shifting and task sharing, and lack of adequate equipment for delivery of needed services were reported as barriers to effective implementation of task shifting ad task sharing. The availability of adapted policies, the political will of the health sector leadership, acceptance of task shifting and task sharing implementation by health workers, and the implementation of actions to improve knowledge and skills of health workers to implement shifted or shared tasks by various actors were the suggested factors promoting the implementation of task shifting and task sharing. Strategies for improving future task shifting and task sharing implementation were improving staffing levels, scaling up trainings and periodic retraining, mentoring and supportive supervision, and improving evidence generation, use and dissemination [31].

### Implementation framework for task shifting and task sharing

The findings of the scoping reviews and the qualitative study were synthesized (Table 1) into an implementation framework. The findings of the scoping review in Africa provided evidence of the contextual factors (scope, rationale and intended goal for task shifting and task sharing) in Africa.

**Table 1:** Synthesis of implementation framework constructs based on scoping reviews and qualitative study findings

Implementation framework constructs	Scoping review – Rationale and scope	Scoping review – Health professions education strategies	Qualitative study
**Context**			
Population needs	The shortage of health workers to meet the health needs of a population is a key rationale for task shifting and task sharing. Other key rationales are to optimally utilize the existing health workers to meet the health service utilization needs and to expand access of a target population to health services.	The provision of training (inservice and preservice) clinical supervision and mentoring, supportive supervision and job aids were to meet the health needs of target populations.	The policymakers reported the rationale for task shifting and task sharing to be the shortage of health workers to deliver quality health services to the population, ensure the availability of a range of health services, improve the health-seeking behaviour of the catchment population, and increase service utilization towards the achievement of universal health coverage.
**Implementation strategies**			
Generate evidence	Evidence on the populations’ health needs is pertinent in planning for the scope of tasks to be shifted or shared. Evidence on the needs to inform scope and competencies is needed to inform the development of training materials, manuals, protocols and aids.	The development of capacity-building materials was informed by evidence from a needs assessment to ascertain the target population/ patient characteristics and provider skills needed to meet populations’ health needs.	Generating contextual evidence on understanding, barriers to implementation, enablers of task shifting and task sharing, and learning from the performance of similar interventions is pertinent in evidence-based planning toward ensuring shared understanding and value.
Map and engage stakeholders	Stakeholders are vital in the planning and implementation of task shifting and task sharing.	Stakeholders were mapped and their views were ascertained on the adequacy of trainings and supervision for shifted/shared tasks.	Inter-cadre rivalry was reported as a barrier to task shifting and task sharing implementation with inadequate knowledge of its importance. The acceptance of task shifting and task sharing implementation by the primary and beneficiary cadres following their involvement in the planning and implementation processes was reported as a facilitator for task shifting and task sharing implementation.
Develop, implement and review the roadmap	Studies in countries reported the implementation of activities in stages to ensure beneficiary health workers implemented the scope of tasks shifted or shared.	Studies reported the implementation of activities sequentially to enhance the capacities of beneficiary health workers to implement shifted or shared tasks.	The implementation of actions to improve the knowledge and skills of health workers to implement shifted or shared tasks by various actors was reported as a facilitator of task shifting and task sharing implementation.
Develop, implement and review national/sub-national policies and strategies	Implementation of task shifting and task sharing was based on contextual health policy direction and strategy for service delivery.	The capacity building of health workers was informed by a broader national/ subnational strategy to apply task shifting or sharing.	The availability of a national policy that was adapted to the contextual needs of the States, and the political will of the health sector leaders to implement the policy and monitor the implementation process were reported as facilitators of task shifting and task sharing.
Inservice training	Health worker cadres that benefitted from task shifting and task sharing were trained on the scopes of tasks shifted or shared.	Inservice training of health workers to enhance their capacity to implement shifted or shared tasks.	Enhancement of knowledge and skills through inservice training was reported as a facilitator and an important strategy for future task shifting and task sharing implementation.
Clinical supervision and mentoring		Onsite clinical supervision to health workers that benefited from task shifting or sharing following trainings to further improve their capacity.	The importance of experienced health workers providing mentoring support to beneficiary cadres of task shifting and task sharing was reported to improve knowledge and skills.
Supportive supervision		Periodic supportive supervision was conducted to improve the capacity of health workers to implement shifted or shared tasks.	Improvement in the knowledge and skills of the beneficiary cadres of task shifting and task sharing through supportive supervision was reported to enable its implementation.
Training Manual		Studies reported the provision of a training manual/ learner’s guide that was developed for use in trainings and as a supportive reference to guide service delivery.	The use of national training manuals for trainings and retraining was reported as a vital strategy in practice.
Job aids		Studies reported the provision of job aids to serve as a quick reference and enhance capacities for task shifting and task sharing implementation	
Booster/ refresher trainings		The conduct of periodic refresher/ booster training following initial training to strengthen the capacities of health workers to implement shifted or shared tasks was reported in reviewed studies.	The conduct of retraining was reported as vital in enhancing the knowledge and skills of health workers to deliver shifted and shared tasks.
Preservice education		The importance of revising the preservice education curriculum to enhance the capacity of beneficiary cadres to implement shared or shifted tasks and preservice training of health workers to enhance their capacity to implement shifted or shared tasks were reported in studies.	
Regulation		Studies reported some cadres had to pass professional competency exams and be certified following trainings to implement shifted or shared tasks.	
Service guidelines		Service protocols/guidelines were developed and provided to beneficiary cadres for reference during service delivery.	
Monitoring, evaluation, review and learning	Studies reported monitoring, evaluating and reviewing the implementation of task shifting and task sharing and using learnings to improve future implementation of activities including trainings, manuals etc.		The importance of investing in data and evidence generation and dissemination was reported as important in tracking the performance of policy and strategy, and evidence-based planning and implementation.
**Intended change**			
Expected outcome	Task shifting and task sharing were implemented to improve health outcomes through improved access to health services.		

Evidence from the qualitative study provided insights on the barriers and promoters of practice, as well as strategies to enhancing task shifting and sharing implementation. The framework was subjected to expert review by a purposeful sample of experts on task shifting and sharing in Africa to gain perspectives on its applicability in their context. The demographic information of the experts is presented in Table 2 and a summary of the responses is presented in Table 3.

**Table 2:** Demographic characteristics of the subject matter experts

Characteristics	Number	Percent
**Gender**		
Male	27	75%
Female	9	25%
**Age**		
Under 18	0	0%
18–24	0	0%
25–34	4	11%
35–44	19	53%
45–54	8	22%
55 and above	5	14%
**Highest level of education**		
Associates degree	1	3%
Bachelors degree	2	6%
Masters degree	23	64%
Doctoral degree	10	28%
**Country of residence**		
Congo	3	8%
Côte d’Ivoire	1	3%
Ethiopia	1	3%
Ghana	1	3%
Kenya	5	14%
Liberia	1	3%
Nigeria	13	36%
Rwanda	2	6%
Switzerland	2	6%
Thailand	1	3%
Uganda	2	6%
United States	1	3%
Zambia	1	3%
Zimbabwe	2	6%
**Years of experience in health services policy, planning or research in Africa**		
Less than 5 years	1	3%
5–10 years	8	22%
11 − 20 years	21	58%
Above 20 years	6	17%

**Table 3:** Summary of responses from subject matter experts

Evaluation domain	Summary of responses by respondents
Context	Yes. The framework is bound by context so I believe contextual factors are considered across implementation strategies. SME, Kenya Yes, it does. The framework addresses the HRH life cycle of HRH in my context starting out with interventions in pre-service and strategies in adapting those already engaged in-service. SME, Nigeria Yes. I have reviewed the framework and I feel that it is relevant in addressing the human resource shortage in health in Africa. The task shifting as it were, is geared towards maximizing the available health human resources. SME, Kenya Yes, it does. The use of contextual needs and evidence to inform the development and translation phases. The framework also addresses the missing elements of institutionalization and implementation of actions in Africa. SME, Nigeria Yes, it does because it might help in formalizing the task shifting and task sharing in countries by handling it in a comprehensive manner rather than just at the programmatic level of priority programmes. SME, Zambia Yes, but will still require more contextualization in each country and even localities in a country. SME, Zimbabwe
Applicability	This framework is well-articulated and comprehensive and has implications for reforming health policies for practice. SME, Thailand The framework is easy to understand and applicable in most African contexts. SME, Nigeria The framework is suitable for task shifting and sharing in Africa. SME, Nigeria Very applicable in light of shortages in the numbers, diversity and ranges of human resources for health in Africa. SME, Uganda This framework is very practical and should be easy to implement in practice. SME, Ethiopia The framework design will use evidence through an iterative process to inform strategy development aligned to the contextual issues. This will ensure that actions are targeted to where they are needed and would have the greatest impact on service delivery, quality, efficiency gains and overall health improvement of the population. SME, Nigeria The implementation framework has the potential to increase the pool of skilled health workers attending primary health care in hard-to-reach communities. With the population increase in Africa and the dearth of skilled health workers exacerbated by health workers’ migration, this framework will be instrumental to achieving universal health coverage. In Nigeria, there is already a demand for the implementation of the TSTS policy, so the framework is applicable. SME, Nigeria The framework provided practical steps and activities that promote the implementation of task shifting and sharing policies and therefore seems widely applicable. SME, United States The framework is very applicable in the practice as it takes into consideration all the elements of an implementation framework and particularly the context at every stage. Signifying Africa’s contextualization and adaptability. SME, Nigeria The context provides the information where the task shifting and task sharing are to be done and further gives room for the understanding and development of the population’s needs. On the above premise, the rest of the framework is brought in simultaneously or concurrently. It is very similar to the established tabular Theory of Change but is very specific to the task it hopes to solve, which is task sharing and shifting. The framework is applicable as it is impressively easy to understand and use. A point in case, this is my first time seeing it, and in less than 5 min I can explain what it means and how it should be used. SME, Nigeria
Revisions to improve the framework	I was thinking you could add short internships as part of the framework unless this is already captured as part of the practicum. SME, Thailand Integration into existing Continuing Professional Development Programs (CPD) to enhance lifelong learning and improvement. SME, Liberia Strong monitoring and evaluation of the implementation framework’s expected outcomes are needed to improve the framework. SME, Nigeria Framework seems great and can be applied where the policy space accepts. SME, Uganda The framework is comprehensive. The implementation strategies should also include how to obtain the government’s commitment to financing the actions and interventions. SME, Nigeria Here are some aspects that could be added to improve the framework: Standardization of training and certification: There is a need to standardize the training and certification of health workers involved in task shifting and task sharing to ensure that they have the necessary skills and knowledge to perform their roles effectively. This could be achieved through the development of standardized curricula, training materials, and certification processes. SME, Rwanda

**Fig. 1:**
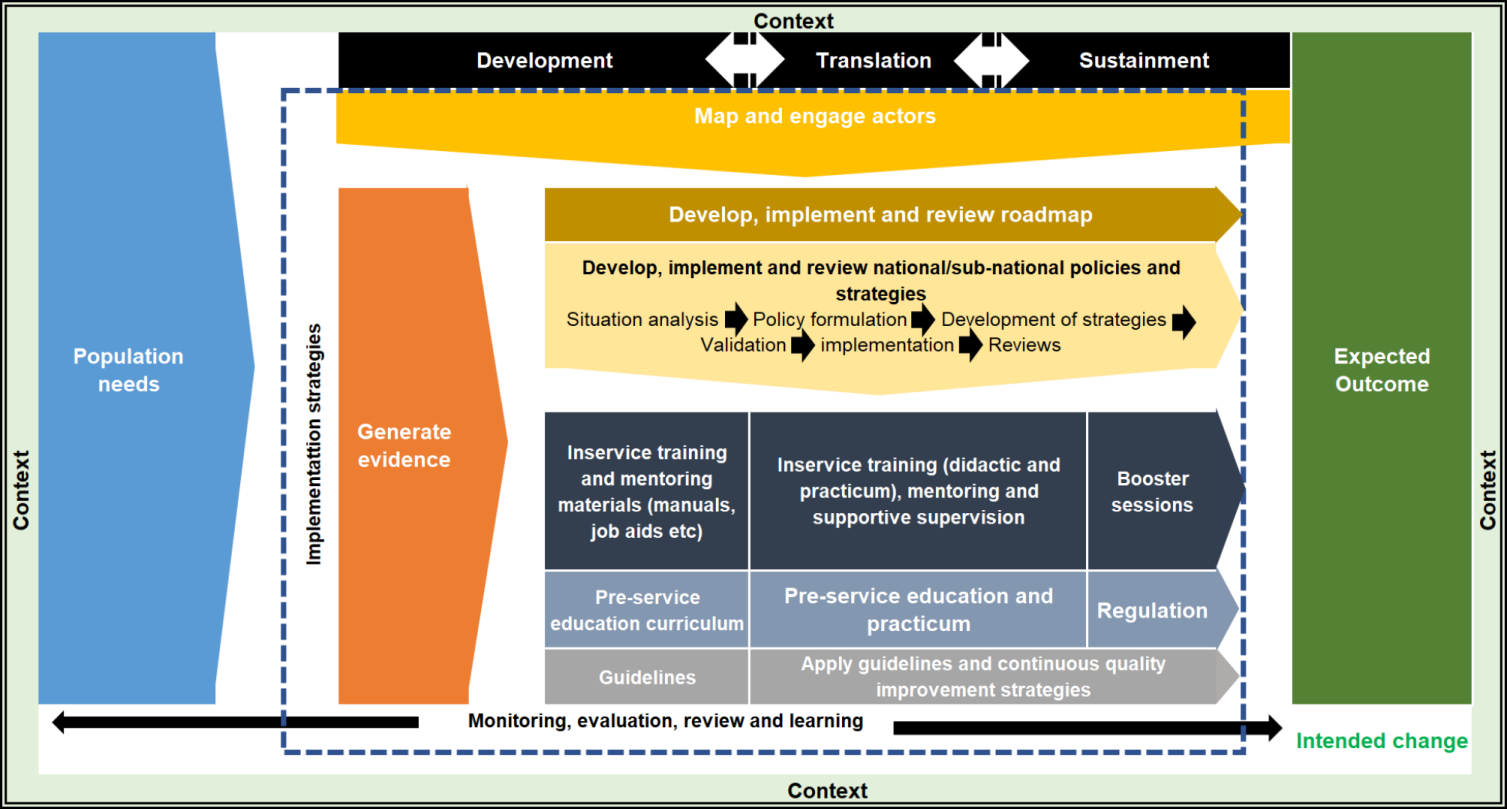
Implementation framework for task shifting and task sharing in Africa

The post-evaluation implementation framework for task shifting and task sharing in Africa is presented in Fig. 1. The core phases of the framework are the development, translation, and sustainment phases, and the core components were intended change, context, and implementation strategies [19]. This framework illustrates the rationale and the role of a combination of factors (enablers and barriers) in influencing the implementation of task shifting and task sharing in Africa towards achieving set national goals. Details on the components and phases are presented below.

### Core component 1 - context

The context focuses on the group of dynamic situational factors or variables that determine or influence the success or otherwise of interventions or approaches towards achieving the intended change (expected outcome) [19, 32]. Therefore, because the context is an essential aspect for consideration at all stages of implementation because they shape the achievement of the intended results, an understanding of the variables is imperative at every stage.

In health service delivery, the context shapes the population’s health needs which informs the leadership and governance actions to ensure that the population access quality health services based on demography, socioeconomic factors, and disease epidemiology. This informed the context being a critical component of the implementation framework. Thus, generating evidence on the contextual factors or variables is vital in ensuring that contextual strategies are implemented to achieve set outcomes and the setting of the expected outcome considers the contextual characteristics.

### Core component 2 - implementation strategies

The implementation strategies refer to interventions or actions, that are based on the needs, and focus towards achieving the intended change (expected outcome). The implementation strategies ultimately result in the intended change and needs to be informed by contextual factors, tailored to fit contextual needs, and be based on contemporary evidence, and rigorous [19, 32].

In the task shifting and task sharing implementation framework, the interventions/ actions are presented in three (3) phases – development, translation and sustainment – as proposed by Huybrechts et al. [19]. These phases are iterative and their adaptation should be based on contextual factors, including barriers and enablers. Its implementation should be informed by guiding principles that include clearly identifying and postulating the actions/ interventions, rationale, target, actors, temporality and planned results/ outcome [32].

#### Development phase

The actions in this phase are preliminary and exploratory but are critical for successful implementation towards sustainability and resilience [19]. They focus on ensuring relevant stakeholders are identified and engaged, a consensus is reached on acceptance of intervention and intended activities, and evidence on contextual variables, including current knowledge, attitudes and practices, are generated for use in the next phase.

Mapping and commencing the engagement of stakeholders in the development phase is vital as it provides insight into the intention to adopt task shifting and task sharing. The mapping should focus on the power, interests and influence of health policymakers, health planners, health worker cadres with service delivery responsibilities in the planned setting, educators, regulatory bodies and professional associations for these cadres, other ministries that have roles in service delivery and health worker management and training etc., within the context. In addition to the mapping, the identified stakeholders should be engaged appropriately, with delegates obtained to form part of the core team to guide and/or lead the implementation process.

Generating evidence on the situation is a vital strategy and the engaged stakeholders should play a huge role in this. The contextual evidence generation process aims to explore and understand the contextual knowledge, attitude, and practices, assess the readiness of the stakeholders and planned setting for the planned intervention. The exploration should also gain understanding on likely barriers and enablers of the task shifting and task sharing implementation and seek to learn from existing interventions to develop bespoke and novel innovative strategies. Furthermore, the evidence generation actions should also include a situation analysis to inform the development of contextual task shifting and task sharing policies and strategies at national or sub-national levels with clear tasks being shifted or shared, and relevant service delivery clinical guidelines. Information on tasks being shifted or shared, based on consensus with stakeholders including educators, will guide the needs assessment and training programme (manual, job aids etc.) and pre-service curriculum development processes [33].

Based on evidence generated and the consensus of stakeholders to implement task shifting and task sharing, a roadmap or action plan should be developed. Roadmap would be developed by stakeholders and should have specific, measurable, achievable, realistic and time-bound objectives, outputs, targets and activities with specified responsible persons and timelines. The monitoring, evaluation, review and learning specifics should also be specified. The activities in the roadmap should cover periodic evidence generation actions, advocacy and communication, task shifting and task sharing policy, strategy and clinical guidelines development, implementation modalities and review, inservice and preservice programme development, training, retraining, and regulation processes. The roadmap should be costed to show the level of investment needed and approved by relevant national authorities to ensure political commitment in its implementation.

The development of inservice training programmes and materials, preservice curriculum and clinical guidelines should also be initiated at this phase. Depending on consensus by the core implementation team and timelines set in the contextual roadmap, these can either be completed at this phase or in the translation phase. They should be informed by evidence generated on contextual factors, align to the regulatory provisions, and tailored to the needs of the population, and the service providers based on shifted or shared tasks.

#### Translation phase

This translation phase focuses on transforming the evidence to practice by integrating the policies, strategies and approaches from the development phase into routine practice in target settings, and monitoring their effect [19, 34]. This is aimed at achieving the intended change by applying actions tailored to the contextual needs, and where necessary, making evidence- based modifications.

The implementation framework proposes the continuous engagement of stakeholders during this phase to ensure buy in and smooth implementation of strategies based on the road map developed in the previous phase. Evidence-based national and/or subnational policies and strategies, with monitoring, evaluation, review and learning plans, should be developed and validated with their implementation started. They are vital in fostering an enabling environment for task shifting and task sharing implementation.

The finalization and/or roll-out of the inservice training programmes and preservice curricula for cadres of focus, and application of continuous quality improvement measures should be conducted in this phase. The inservice trainings, with didactic and practicum sessions, should be targeted at the health service providers to enhance their capacities to implement shifted or shared tasks. They should be based on the finalized clinical guidelines specifying the tasks, with relevant training manuals and job aids provided. Also, mentoring, clinical supervision and supportive supervision by experienced health workers should be instituted based on pre-defined protocols. For the preservice training, the contextual curricula for relevant cadres should be rolled out with practicum also incorporated to enhance their skills. Regulation practices for the cadres of focus should also be enhanced to ensure that accreditation mechanisms incorporate measures for ensuring needed educators, infrastructure and equipment are in place.

#### Sustainment phase

The focus of the sustainment phase is to sustain and institutionalize strategies from the development phase to maintain the intended change. This ensures that continuous improvement is institutionalized, with pertinent actions becoming a part of daily practice [19].

The engagement and views of the stakeholders is vital in this phase as they provide insights on how practice has fared based on the status of the monitoring indicators in the translation phase. They are also vital in the review and evaluation processes in the roadmap, and the policies, strategies and guidelines, as well as the inservice and preservice education. These review and evaluation processes are vital in designing contextual continuous improvement strategies that are pertinent for sustainment and institutionalization upon implementation.

The conduct of booster sessions of inservice trainings for health workers is another important action in this phase. This ensures that contemporary knowledge is disseminated, and skills are further enhanced. This also applies to continuous clinical mentorship and periodic supportive supervision. For preservice education, strengthening practicum experiences based on contemporary trends and regulation ensures that graduates have the requisite skills to deliver health services on graduation.

### Core component 3 - intended change

The intended change refers to the consciously planned innovation, change and/or result (output, outcome or impact) expected based on interventions implemented to achieve the set objective(s)[19, 35]. In the proposed framework, the intended change is the expected outcome of the task shifting and sharing practice. Several expected outcomes have been suggested in various contexts, including optimal utilization of existing health workers for delivery of quality health services within particular settings [10, 36–38] [39–41] and expanding access to select health services to other levels of care and geographical locations [42] [9] [43] [44].

## Discussion

This study presented an implementation framework for task shifting and task sharing based on findings of studies in Africa. To develop the framework, a sequential multimethod research design was applied in phases. Two scoping reviews were conducted to synthesize evidence on the rationale and scope of task shifting and task sharing in Africa, and the health professions education strategies applied to enhance capacities for task shifting and task sharing implementation in Africa. A qualitative study exploring the perceptions of policymakers on the barriers, promoters, and strategies for improving task shifting and task sharing implementation in Nigeria was also conducted. The evidence generated from the scoping reviews and the qualitative study was synthesized into an implementation framework that was expertly reviewed by subject matter experts who evaluated the applicability of the framework in Africa. We opine that this implementation framework is applicable in other contexts based on the rigour employed during its development.

Implementation frameworks to guide actions aimed at achieving national, regional and global goals are vital in bridging the gap that exists between research evidence and public health practice [45]. They are also essential in facilitating the apposite use of health research findings in policy and practice by policymakers, and health managers, thereby reducing the use of personal ideas in solving health sector issues [16, 17]. For task shifting and task sharing, considering its widespread application and ongoing expansion of the scopes of practice of some cadres of health workers, an implementation framework, based on contemporary evidence which is adaptable, is vital in ensuring actions can be taken within a short space of time to ensure goals are achieved.

This study, to the best of the authors’ knowledge, is the first study that has attempted to develop an implementation framework for task shifting and task sharing based on findings within the African context which faces numerous health workforce and service delivery challenges. This study, therefore, provides an imperative implementation framework that can be adapted to other service delivery practices in various settings. The framework is quite comprehensive and highlights the core components to be considered and provides a chronological iterative guide of factors to be considered and phased strategies that should be applied to initiate relevant processes and generate contextual evidence, translate evidence into policy and practice, and implement health systems strategies to sustain practice and intended change.

A core component of the proposed implementation framework is the context (or setting). In policy and practice, the context plays a vital role in ensuring that interventions are fit–for–purpose, and are successful [19]. The importance of assessing context for strategies to be successful, address inequalities, and use the findings to improve policy and practice is evident in the literature [19, 46, 47]. Considering that the context shapes the health needs of a population in the development and emergency contexts [48, 49], its exploration is important in ensuring that planned objectives are achieved. Therefore, taking into cognizance the context is critical in the implementation of task shifting and task sharing, across all phases and in all actions. For policy and practice, evidence on the contextual factors should be obtained and used in developing and implementing tailored strategies to achieve expected outcomes.

The second core component of the framework, the implementation strategies, presents iterative actions in three phases (development, translation, and sustainment) that are aimed at achieving the intended change (expected outcome) that is based on the contextual objective of the task shifting and sharing practice. The strategies in the framework include mapping and engagement of stakeholders, generating evidence on contextual factors, and development, implementation and review of a road map (or action plan) and national and/or sub-national policies and strategies. Others include the education of health workers using context-based manuals, job aids, curriculum and guidelines, regulation of practice and education, and monitoring, evaluation, review and learning.

Identification and engagement of all relevant stakeholders are important for the successful delivery of any intervention as their involvement is vital in ensuring ownership, political will and active participation [50, 51]. Considering the aforementioned, this strategy is recommended in all phases of the framework. Also, evidence generation on contextual factors is indicated to inform the policy and strategy to ensure enabling environment and political will [52, 53]. Evidence is also crucial in the shaping of health worker education materials and strategies apposite for quality training and service delivery [9, 54]. Other suggested strategies, including clinical mentoring periodic supportive supervision, and provision of training manuals, clinical guidelines and job aids have been reported in other studies as important [11, 55–59]. The continuous holistic monitoring, evaluation, review and learning process is essential in ensuring agreed actions are being taken, targets are being met, course-correction strategies are implemented on time and the intended change is attained [60, 61].

The third core component, intended change, varies widely by programme and context. Although this has been reported to include optimal utilization of existing health workers [10, 36–38] [39–41] and expanding access of the population to health services [42] [9] [43] [44], within particular settings, it is vital that this is clearly articulated as it will inform the strategies that will be set.

### Implications for policy and practice

The implementation framework for task shifting and task sharing in Africa, which is adaptable to other contexts, provides a critical path for undertaking actions to achieve set objectives with contextual evidence translated into practice. The implementation framework leans strongly on context as it is a critical aspect of programmes and interventions. This study further portrays its importance in all stages of task shifting and task sharing implementation and practice. Thus, its understanding and use in tailoring task shifting and task sharing policy directions, strategies and practice actions are essential. The role of the various contextual stakeholders, within and outside the health sector, in achieving the aforementioned was also evident from this study and is also vital in policy, strategy and guideline development, implementation and review. Also, evidence from this study is the importance of generating contextual evidence and using them to apply a wide array of approaches to improve the knowledge and skills of existing and future health workers to deliver integrated – people-centred health services within particular contexts. To track progress and ensure set objectives are achieved, monitoring, evaluation, reviews and learning, must be a strong part of the planning, development and implementation stages.

### Limitations

Despite this study and the implementation framework being novel, we acknowledge some limitations. The scoping reviews focused on peer-reviewed literature and we may have missed the task shifting and task sharing practices that are documented elsewhere. Also, whilst the scoping review focused on three databases, studies that are available in other databases may have been missed. The qualitative study setting was Nigeria with Africa being large and its health service contexts being diverse. Perhaps, a multi-country approach to the qualitative component would have unraveled a wide array of contextual factors. Whilst we advocate for further research in other countries, we feel that the scoping reviews provided substantial insight into contextual factors that apply in other countries in Africa.

## Conclusions

The implementation framework for task shifting and task sharing in Africa serves as a guide on actions needed to achieve national, regional and global goals based on contextual evidence. The framework has three core components – context, implementation strategies and intended change. The implementation strategies comprise of iterative actions in the development, translation, and sustainment phases that to achieve an intended change. The implementation strategies in the framework include mapping and engagement of stakeholders, generating evidence, development, implementation and review of a road map (or action plan) and national and/or sub-national policies and strategies, education of health workers using manuals, job aids, curriculum and clinical guidelines, and monitoring, evaluation, reviews and learning.

### Electronic supplementary material

Below is the link to the electronic supplementary material.

Supplementary Material 1

## Data Availability

The datasets used and/or analysed during the current study available from the corresponding author on reasonable request.
