# Supplementary material for: Optimizing the roles of health workers to improve access to health services in Africa: an implementation framework for task shifting and sharing for policy and practice
Source: BMC Health Serv Res. 2023 Aug 9;23:843. doi: 10.1186/s12913-023-09848-z (PMC10410914; doi:10.1186/s12913-023-09848-z)
Supplement: Supplementary file 1 — Supplementary Material 1 [file 12913_2023_9848_MOESM1_ESM.pdf]

IMPLEMENTATION FRAMEWORK REVIEW FORM

1.	Does the implementation framework address relevant **Context** information for Africa/ Nigeria?
2.	Provide your opinion on the **Applicability** of this framework in practice or otherwise?
3.	What aspects need to be added to improve the framework
